# Asiatic acid alleviates Ang-II induced cardiac hypertrophy and fibrosis via miR-126/PIK3R2 signaling

**DOI:** 10.1186/s12986-021-00596-7

**Published:** 2021-07-13

**Authors:** Haiyu Li, Xiaoxu Tian, Yongjuan Ruan, Junhui Xing, Zhe Meng

**Affiliations:** Department of Cardiology, The First Affiliated Hospital of Zhengzhou University, Zhengzhou, Henan China

**Keywords:** miR-126, PIK3R2, PI3K/Akt signaling pathway, Cardiac hypertrophy

## Abstract

**Background:**

Cardiac hypertrophy is an independent risk factor of many cardiovascular diseases. Studies have demonstrated that microRNA-126 (miR-126) was involved in angiogenesis during physiological and pathological process. However, its role in cardiac hypertrophy has not been known clearly. Our previous study demonstrated that asiatic acid (AA) has obvious protective effect on cardiac hypertrophy. Here, this study aimed to discover the regulatory role of miR-126 and its mechanism in cardiac hypertrophy, and to determine whether AA’s anti-hypertrophy effect is partially miR-126 dependent.

**Methods:**

Male Sprague Dawley rats were AngII infused via osmotic minipumps for 4 weeks and were treated with AA (20 mg/kg/day) by oral gavage. Cardiac hypertrophy was assessed using the echocardiography and histological analysis. In vitro studies,cardiomyocyte and cardiac fibroblasts (CF) were treted with AngII and AngII plus AA. And, the effect of AA on miR-126 and PI3K/AKT signaling pathway was investigated.

**Results:**

Treatment of rats with AA decreased the ratio of heart weight to tibia length and hypertrophy markers. In vitro exprements demonstrated that AA significantly attenuated AngII-induced cardiac growth and cardiac fibroblast collagen expression. Moreover, our results found downregulation of miR-126 and activation of PI3K/AKT signaling pathway in AngII infusion induced cardiac hypertrophy model. It was also determined that miR-126 targets PIK3R2 directly.

**Conclusions:**

AA supplementation upregulated the expression of miR-126 and conferred cardio-protection effect against AngII induced cardiac hypertrophy.

## Background

Globally, cardiovascular disease (CVD) remains the leading cause of mortality and morbidity [1]. Cardiac hypertrophy is one of the most important stage during the development of CVD, but its mechanism has still not fully been understood. Pathological cardiac hypertrophy is now recognized as an independent risk factor for heart disease, including coronary artery disease (CAD), arrhythmia, heart failure, hypertension and even sudden death [2].

Asiatic acid (AA), a natural triterpenoid compound extracted from *Centella asiatica*, has been identified as a potential therapeutic agent as it demonstrates anti-hyperlipidemia, anti-inflammation [3], anti-fibrosis [4] and anti-oxidation effect [5]. Previous studies have reported that AA ameliorates liver fibrosis through regulating the PI3K/AKT/mTOR signaling pathway [6]. Our recent study demonstrated that AA attenuates cardiac fibrosis and improves cardiac function via regulating TGF-β1/Smads signaling pathway [7].

MicroRNAs (miRNAs), a kind of small non-coding RNAs containing approximately 22 nt [8], play important roles in almost all of physiology and pathophysiology process, including proliferation, apoptosis as well as fibrosis [9–11]. They serve as post-translational regulators and negatively regulate gene expression by binding to their complementary sequence within their target mRNAs 3’ untranslated regions (UTRs), leading to mRNA degradation or blocked translation [12]. Recently, the crucial role of miRNAs in cardiac function regulation has attracted much more attention [13, 14]. Interestingly, miR-126 was reported to play a critical role in angiogenesis by activating mitogen-activated protein kinase (MAPK) and PI3K/AKT signaling pathway [15, 16]. Previous studies have reported the PI3K/AKT signaling pathway was involved in the development of cardiac hypertrophy and fibrosis. miR-126 can regulate PI3K/AKT signaling pathway by targeting PIK3R2 (PI3K regulatory subunit 2) [19] and affect the proliferation, migration and angiogenesis.

In the present study, we aimed to explore the regulatory role of miR-126 in cardiac anti-hypertrophy after AA treatment. Using AngII infusion induced hypertrophy model, we showed for the first time that AA alleviates cardiac hypertrophy is associated with miR-126/ PI3k/AKT signaling pathway. PI3k/AKT pathway was inactivated and regulated by miR-126. Our results might help to deepen the understanding of the role and function of miRNA-126 in cardiac hypertrophy. These findings offer important insights into fundamental mechanisms underlying functions of AA and miRNA, meanwhile, would provide a potential therapeutic targets for cardiac hypertrophy.

## Methods

### Materials

Asiatic acid (C_30_H_48_O_5,_ MW: 488.70) was purchased from Guangxi Changzhou Natural Products Development Co. Ltd (> 95% purity, Guangxi, China). Antibiotic–antimycotic solution (10,000 units/ml of penicillin, 10,000 ug/ml of streptomycin) Ang II was obtained from Sigma Chemical Co. (St Louis, MO, USA). Antibodies against ANP, β-MHC, PIK3R2, PI3K, p-PI3K, Akt, p-Akt, GAPDH were purchased from Abcam CO (Cambrige, UK).

### Animal

8-week-old male Sprague Dawley (SD) rats (150–170 g body weight) were purchased from Beijing Vital River Laboratory Animal Technology Company (Beijing, China). All experiments involving rats were approved by the Institutional Animal Care Research Advisory Committee of the National Institute of Biological Science (NIBS) and Animal Care Committee of Zhengzhou University. All rats were maintained on a 12:12-h light–dark cycle and have free access to water and food.

### Experimental design and treatment protocol

A rat model of AngII infusion induced cardiac hypertrophy was established. In brief, SD rats were quickly anesthetized with an intraperitoneal injection of sodium pentobarbital (50 mg/kg), then the prefilled osmotic minipumps (Alzet, Model 2002) were implanted into the subcutaneous tissue to deliver AngII (Sigma-Aldrich, A9525) at 400 ng/kg/min for 4 weeks. Rats were randomly divided into two groups (10 mice/group) for different treatments by oral gavage for 4 weeks: AngII group and AngII + AA (AA 20 mg/kg) group. Another ten SD rats as the wild control group sham group.

### Cell culture

Rat cardiomyocyte H9c2 cells cardiac fibroblasts (CFs) were cultured in DMEM with 15% FBS, 100 U/mL penicillin and 100 μg/mL streptomycin in a humidified atmosphere of 5% CO_2_ and 95% air at 37 °C. Cells were incubated with AA 160uM with or without AngII 10uM for 24 h in a 6 well plate. Cell surface area analysis was performed using confocal microscopy as described previously [20].

### Echocardiographic study

Transthoracic echocardiography was performed to access left ventricular (LV) function variables. Briefly, after the induction of general anesthesia, rats were placed in a supine position. Rats were underwent transthoracic two dimensional guided M-mode echocardiography with a 12L MHz transducer (Sibiscape Co. Ltd.). From the cardiac short axis, the LV anterior wall end-diastolic thickness (LVAWd), the systolic LV anterior wall thickness (LVAWs), the LV internal dimension at end-diastole (LVIDd), the LV internal dimension at end-systole (LVIDs), the LV posterior wall end-diastolic thickness (LVPWd), the LV posterior wall end-systolic thickness (LVPWs), the ejection fraction (EF), and fractional shortening (FS) were measured.

### Histological analysis

The left ventricle were fixed in 10% formalin and embedded in paraffin. Tissue Sections (4 µm) were stained with 1.0 mg/ml Alexa Fluor 488® conjugate of wheat germ agglutinin (WGA) solution (MolecularProbes, Eugene, OR, USA) to demonstrate the size of cardiomyocytes. The collagen deposition in the left ventricle was detected by Masson staining. Ten fields in each region of the heart were selected randomly from four nonconsecutive serial sections, and collagen content was quantified by measuring the total blue area per square millimeter using the ImageJ.

### Dual-luciferase gene reporter assays

Based on the PIK3R2-wild type (wt), a complementary sequence mutation site of the seed sequence was designed as mutant type (mut). PIK3R2 wt and mut were amplified and cloned into a pGL3 vector containing the firefly luciferase reporter gene (ObiO, Shanghai, China). Human embryonic kidney (HEK) 293 cells were co-transfected with 100 ng of recombinant luciferase reporter plasmid, 10 ng of pRL-TK as an internal control, 50 nM miR-126-3p mimic, or mimic control respectively. Luciferase activity and renilla luciferase were measured 24 h after transfection using a dual-luciferase reporter assay. And the ratio of firefly luciferase/renilla luciferase was calculated and wasused to reveal the interactions between miR-126 and PIK3R2.

### Western blotting

At the end of experiment, rats were sacrificed and the heart were quickly removed. Left ventricle tissue were lysed by RIPA lysis buffer and phenylmethylsulfonyl fluoride (Beyotime, China). The protein concentration was detected by using a BCA protein assay kit. Equal amounts of protein (30 μg) were separated using 10 or 12% SDS-PAGE and were transferred onto a polyvinylidene difluoride membrane (PVDF). Next, PVDF membranes were blocked with 5% fat-free milk and incubated with primary antibodies overnight at 4℃. Subsequently, the membranes were washed and incubated with secondary antibodies at room temperature. The optical density of the bands was visualized by an ECL system (Pierce). GAPDH was used as an endogenous control. Data was normalized to GAPDH levels.

### RNA isolation and quantitative real-time PCR

Total RNA was extracted from the frozen tissues or treated H9c2 cells and CFs using Trizol reagent (Invitrogen, USA). First strand cDNA was synthesized using an RT kit (Invitrogen, USA). miRNA from heart was converted to cDNA using the miRNA 1st Strand cDNA SynthesisKit (Vazyme, Nanjing, China) according to the manufacturer’s protocol. Amplifications were performed using an opticon continuous fluorescence detection system with SYBR green fluorescence (Molecular Probes, Eugene, USA). A single melting curve peak confirmed the presence of a single product. Results were expressed as fold differences relative to GAPDH or U6 using the 2-ΔΔCT method. All the primers were synthesized by Sangon Biotech (Shanghai, China) and the sequence are listed in Table 1.

**Table 1:** Primers used for reverse transcription and real-time PCR

Primer names		Sequences
ANP	Sense Anti-sense	CCTTCTCCATCACCAA TGTTATCTTCGGTACCG
PIK3R2	Sense Anti-sense	ACTCACCTTCTGCTCCGTT TCTGGTCCTGCTGGTATTTGG
U6	Stem-loop Sense Anti-sense	GTCGTATCCAGTGCAGGGTCCGAGGTATTCGCACTGGATACGACAA AGAGAAGATTAGCATGGCCCCTG ATCCAGTGCAGGGTCCGAGG
miR-126	Stem-loop Sense Anti-sense	GTCGTATCCAGTGCAGGGTCCGAGGTATTCGCACTGGATACGACCGCATT CGCGTCGTACCGTGAGTAAT AGTGCAGGGTCCGAGGTATT
pri-miR-126	Sense Anti-sense	AAGGACCATTGTTGGCACTCAGG TCTCACCACGCTGTCCACTCC
β-MHC	Sense Anti-sense	GCCGAGTCCCAGGTCAACAA GTAATTCGAGGGCAGGAACCC
Co1 I	Sense Anti-sense	ACTCAGCCCTCTGTGCCT CCTTCGCTTCCATACTCG
Co1 III	Sense Anti-sense	AGATGCTGGTGCTGAGAAG TGGAAAGAAGTCTGAGGAAGG
GAPDH	Sense Anti-sense	GACATCAAGAAGGTGGTGAAGC TGTCATTGAGAGCAATGCCAGC

### Statistical analysis

All data are presented as means ± SEM. SPSS 21.0 was used to perform statistical analysis of the data. Statistical differences were calculated with the 2-tailed Student t test when comparing 2 conditions, and ANOVA was used when comparing > 2 conditions. A value of P < 0.05 was considered statistically significant.

## Results

### AA attenuated AngII-induced cardiomyocyte growth and CF collagen expression in vitro

**Fig. 1:**
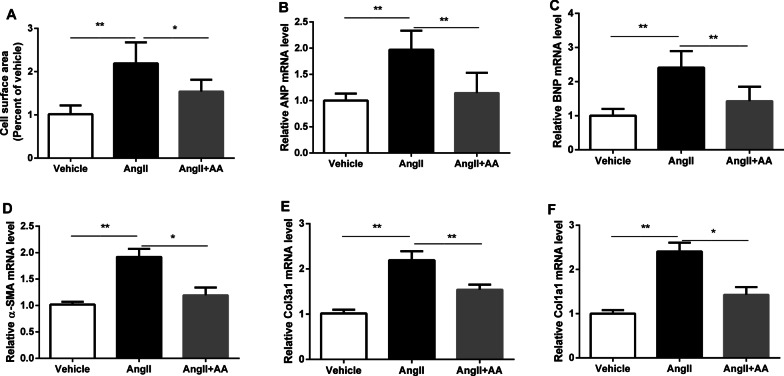
Asiatic Acid (AA) treatment inhibited AngII-induced cardiomyocytes growth and cardiac fibroblasts collagen expression in vitro. H9c2 and cardiac fibroblasts (CF) cells were exposed to AngII 10 µM with or without AA 160 µM for 24 h. Cell surface area was determined (**a**), mRNA level of ANP (**b**) and BNP (**c**) were quantified using real-time PCR. **c** α-SMA, Col1a1 and Col3a1 mRNA levels in AngII-induced and AA-treated CFs. Data are presented as mean ± SD, *P < 0.05, **P < 0.01

To investigate whether AA has a protective role on the development of cardiac hypertrophy, cell surface area and hypertrophic markers were assessed in H9c2 cells treated with AngII. Our results demonstrated that hypertrophic markers (ANP and BNP) and cell surface area were significantly increased in H9c2 cells treated with AngII 10 μM for 24 h compared to the control group (Fig. 1a–c). AA treatment was able to significantly inhibit the hypertrophic growth of cardiomyocytes and ANP and BNP mRNA levels in H9c2 cells following 24 h of AngII administration (Fig. 1a–c). In addition, following AngII administration, the fibrotic markers α-SMA, Col1a1 and Col3a1 gene expression were increased in cardiac fibroblasts (CFs), and AA treatment significantly alleviated the increased expression of fibrotic markers (Fig. 1d–f).

### AA ameliorated AngII-induced cardiac hypertrophy and fibrosis

**Fig. 2:**
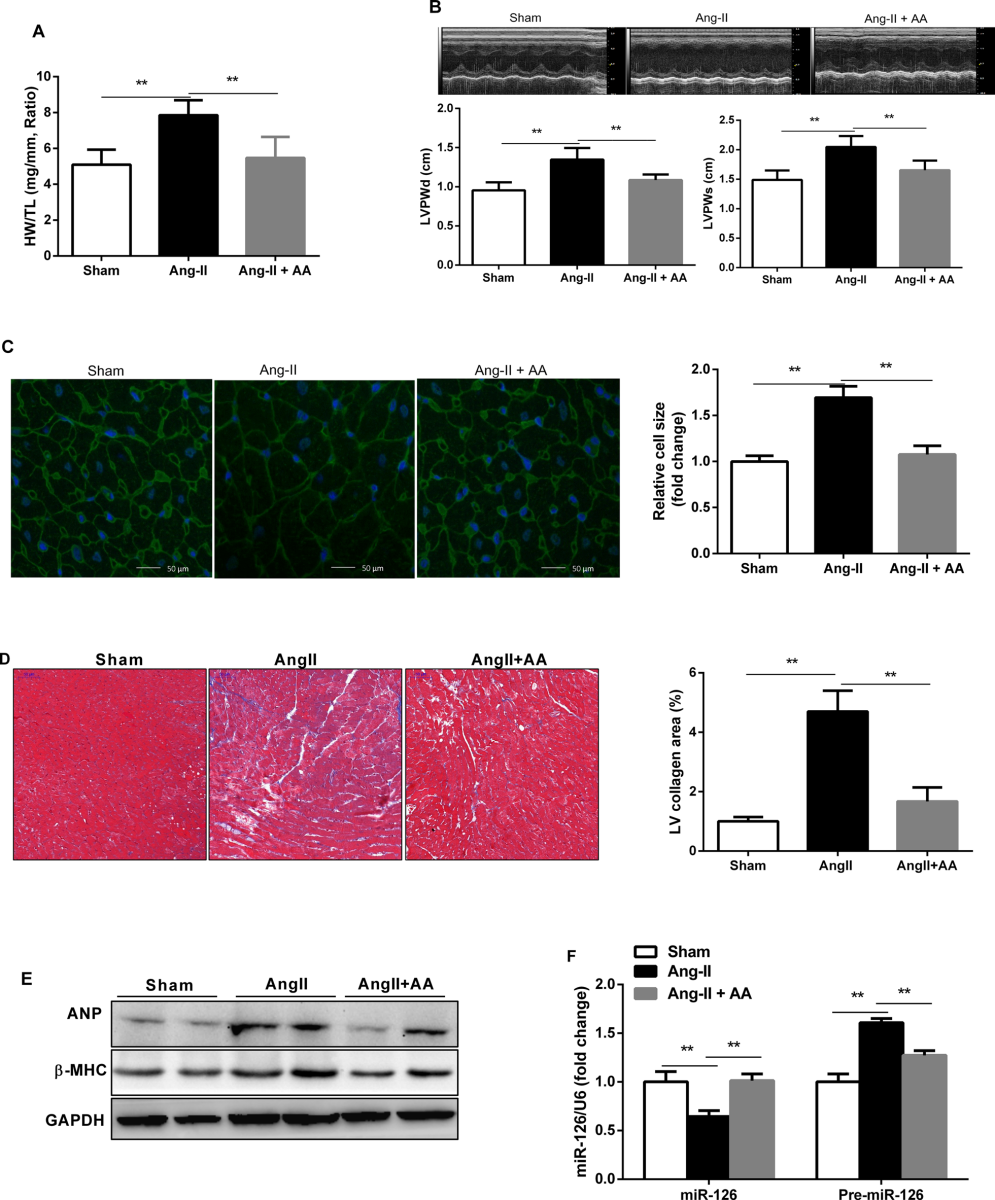
Asiatic Acid (AA) ameliorated AngII-induced cardiac hypertrophy and fibrosis in vivo. **a** Heart weight to tibia length ratio of different groups, n = 9. **b** Representative M-mode echocardiographic tracings of different groups. **c** Representative wheat germ agglutinin-stained of the left ventricles to cardiomyocyte size and quantification of the cardiomyocyte size in the indictated groups (n = 9 per group). **d** Representative Masson-staining of the left ventricles to assess cardiac fibrosis and quantification of the fibrosis area in different groups (n = 9 per group). **e** ANP (atrial natriuretic peptide) and B-MHC protein levels in the heart detected by Western blotting in different groups (n = 6 per group). **f** The expression of miRNA-126 and pre-miR-126 levels in the hearts of the different groups (n = 7 per group). Data are presented as the mean ± SD, **P < 0.01

An animal model of cardiac hypertrophy was established in rats. We measured the ratio of heart weight to tibia length (HW/TL) and evaluated cardiac function by echocardiogram. AngII infusion rats showed a significant increase in the ratio of weight/tibia length (HW/TL), and this increase was attenuated in AA-treated rats (Fig. 2a). The thickness of the left ventricular post wall at the end-diastole (LVPWd) and the end-systole (LVPWs) was higher in AngII infusion rats, while AA treatment attenuated these alterations (Fig. 2b). Results of WGA staining showed that the cell size of cardiomyocytes was significantly increased in the myocardium of the Ang-II infusion animal model, which was significantly attenuated in AA-treated rats (Fig. 2c). To determine the effect of AA on cardiac fibrosis, heart sections were stained with Masson’s staining. In Fig. 2d, interstitial fibrosis is demonstrated by the blue areas. Quantitative data revealed increased collagen deposition in AngII-induced rats, while was significantly attenuated in AA-treated rats (Fig. 2d). Meanwhile, significant increase of ANP and β-MHC protein expression was observed in the hypertrophic rat myocardium, while their expression was inhibited in AA-treated rat (Fig. 2e). Collectively, AA treatment can inhibit AngII-induced cardiac hypertrophy and fibrosis.

### AA promote the expression of miRNA-126 in the hypertrophic AngII-infused rats

To explore novel mechanisms underlying the anti-hypertrophy effects of AA, we focused on miR-126 which has attracted a lot of attention in angiogenesis. Furthermore, we investigated whether miR-126 could contribute to cardiac hypertrophy. The results of qRT-PCR showed that mature miR-126 expression was remarkably decreased whereas unprocessed pri-miRNA-126 was increased in AngII infusion rats compared with control rats (Fig. 2e), and AA treatment elevated the levels of miR-126 while decreased that of pri-miRNA-126 (Fig. 2e).

### AA suppress expression of PIK3R2 and PI3K/Akt signaling pathway in hypertrophic AngII-infused rats

**Fig. 3:**
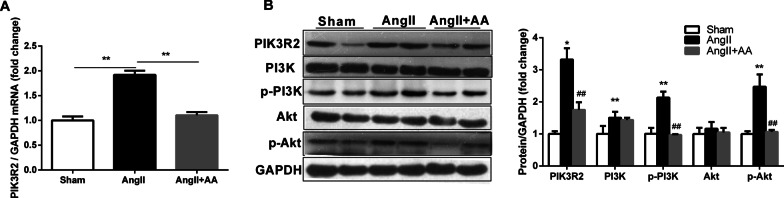
Asiatic Acid (AA) downregulated PIK3R2 expression and PI3K/Akt signaling pathway in AngII-induced cardiac hypertrophy and fibrosis. **a** PIK3R2 gene expression detected by RT-PCR in different groups. **b** PIK3R2, PI3K, p-PI3K, Akt, p-Akt expression levels in the heart detected by Western blotting and quantification of the expression levels in different group (n = 6). Data are presented as the mean ± SD, *P < 0.05, **P < 0.01 for AngII versus Sham, ^##^*P* < 0.01 for AngII + AA versus AngII

Real-time PCR and western blotting analysis were performed to investigate the expression of PIK3R2 mRNA and protein level. Compared with the sham group, the expression of PIK3R2 mRNA and protein were significantly increased in Ang-II infusion rats (Fig. 3a, b). We further determined whether the PI3K/Akt signaling pathway was activated in hypertrophy myocardium. Our results demonstrated that PI3K and Akt phosphorylation increased in Ang II-infusion rat group (Fig. 3b). And AA treatment significantly attenuated PIK3R2 upregulation and PI3K/Akt signaling pathway activation (Fig. 3b). There was no significant difference in total PI3K and total Akt between the control group and Ang-II infusion groups (Fig. 3b).

### PIK3R2 is a target gene of miR-126

**Fig. 4:**
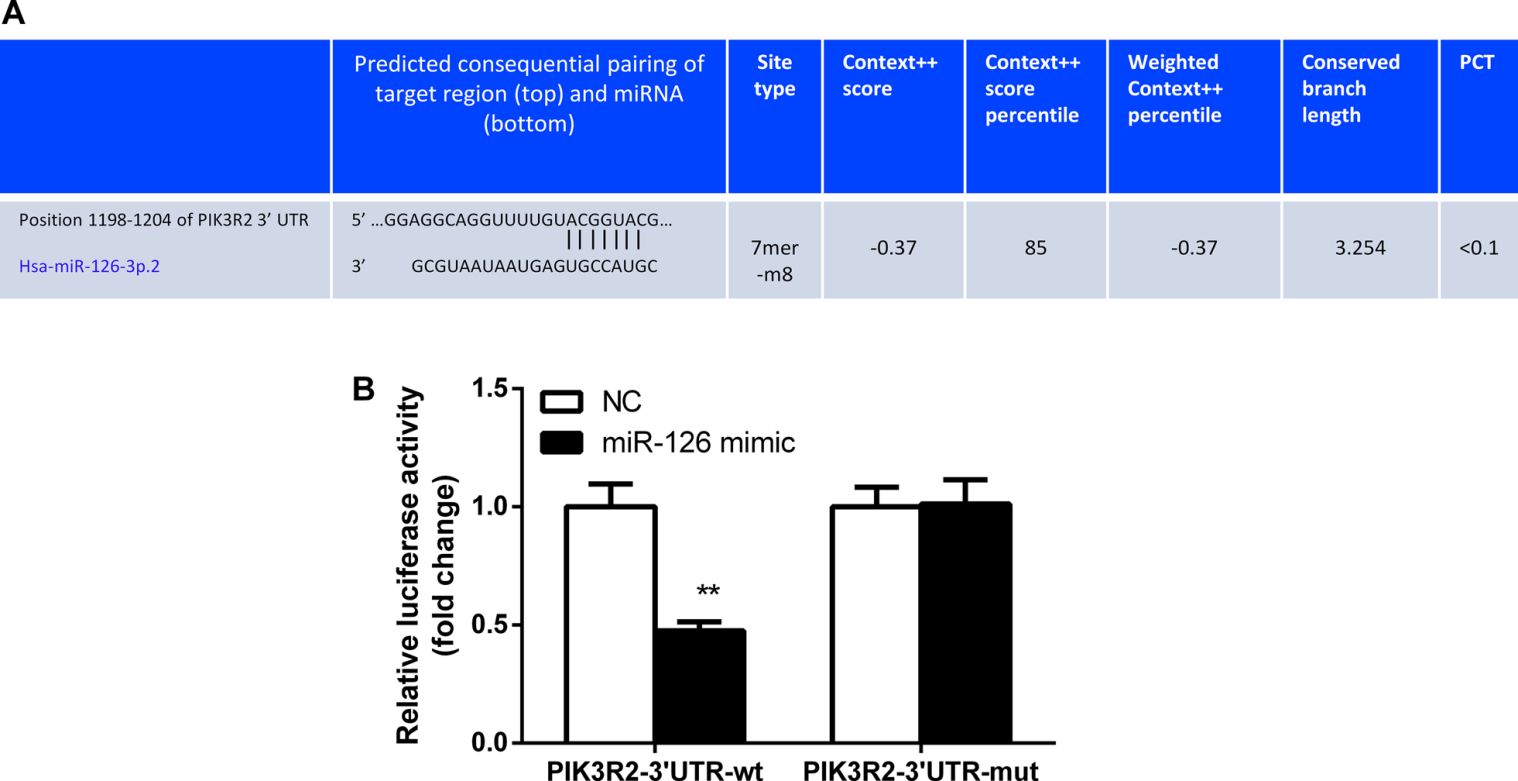
PIK3R2 is a target gene of miRNA-126. **a** The predicted binding sites of miRNA-126 on the 3’UTR sequence of PIK3R2 of PIK3R2 gene; **b** The luciferase activity of PIK3R2-3’UTR-wt and PIK3R2-3’UTR-mut; *P < 0.05 versus the NC group; UTR, untranslated region; wt, wild type; mut, mutant type; NC, negative control. The results of luciferase activity were regarded as measurement data, presented by mean ± SD and analyzed using the t test and the experiment was repeated 3 times

Considering the expression of miR-126 is inversely related with that of the PIK3R2, it seems reasonable to speculate that miR-126 could directly regulate PIK3R2 expression. TargetScan were used to search for the targets of miR-126. The putative target sites for binding of PIK3R2 and miR-126 is shown in Fig. 4a. Mutation was constructed to the putative target sites on the 3’-UTR of PIK3R2. Dual luciferase reporter gene assay was performed to further verify the potential interaction of miR-126 with the 3’-UTR of PIK3R2. Results revealed that compared with the NC group, overexpression of miR-126 significantly inhibited the luciferase activity of PIK3R2-3’-UTR-wt, while exerted no obvious effect on luciferase activity of the PIK3R2-3’-UTR-mut (Fig. 4b). Taken together, miR-126 could specifically bind to PIK3R2-3’-UTR and down-regulate PIK3R2 gene expression at the post-transcriptional level. So, in this section, we confirmed that PIK3R2 is a target of miR-126.

## Discussion

Cardiac hypertrophy and fibrosis caused by multiple diseases including hypertension and heart valvular disease, have become a major cause of morbidity and mortality for people from both the developed and the developing countries. A better understanding of the mechanisms underlying cardiac hypertrophy and fibrosis is important for developing more effective diagnostic and therapeutic strategies. In the present study, we found that miRNA-126 were markedly downregulated in the myocardium of AngII-infused rats, PIK3R2 is a target of miRNA-126, and AA supplementation was able to prevent pathological cardiac hypertrophy and fibrosis by upregulation of miR-126 and inhibition of PI3K/AKT signaling pathway.

AA has been proved to inhibit renal fibrosis and left ventricular remolding. In our previous study, we found AA could inhibit AngII-induced proliferation of CFs and the pressure overload-induced cardiac hypertrophy and fibrosis. Here, an important finding of our study is that AA protected the heart from hypertrophy and fibrosis in response to AngII through upregulation of miR-126 and activation of PI3K/AKT signaling pathway. Our study is the first to show that AngII-induced cardiac hypertrophy and fibrosis is associated with decreased expression of miRNA-126.

miRNAs regulate gene expression at the transcriptional or translational level by binding to the 3’UTR of mRNAs, and affect a variety of cellular pathways. Previous studies have reported associations of miR-126 with angiogenesis [21], atherosclerosis [22] and tumorigenic process [23]. Recently, a study showed that reduction of miRNA-126 drove lung fibrosis by activating the PI3K/AKT/mTOR pathway after carbon black treatment [15]. We found a decrease level of miRNA-126 and an increase level of PIK3R2 in rat cardiac tissue in response to AngII infusion. Considering the expression of miR-126 is inversely related with that of the PIK3R2, we speculated that miR-126 could directly regulate PIK3R2 expression. In our study, we confirmed that PIK3R2 is a target of miR-126, and this finding is consistent with previous studies [15].

Many studies reported that PI3K/AKT signaling pathway was involved in the development of fibrotic diseases, such as pulmonary fibrosis [24], liver fibrosis [6] and cardiac fibrosis [25]. We also found that PI3K/AKT signaling pathway was activated in AngII-infusion rats, while those effects were suppressed by AA supplementation.

## Conclusions

In this study, we arrived at a conclusion that miR-126 targeting PIK3R2 activating the PI3K/AKT signaling pathway, and promoted cardiac hypertrophy and fibrosis in AngII-induced rats. Our study supports the notion that upregulation of miR-126 or inhibition of PI3K/AKT signaling pathway can represent a novel target for future development of therapeutic strategies for cardiac hypertrophy and fibrosis. Therefore, AA supplementation may be considered as potential prevention strategy for cardiac hypertrophy and fibrosis.

## Data Availability

All data generated of analyzed during this study are included in this published article or are available from the corresponding author on reasonable request.

## Abbreviations

AA: Asiatic acid
SD: Sprague Dawley
CVD: Cardiovascular disease
CAD: Coronary artery disease
UTRs: Untranslated regions
MAPK: Mitogen-activated protein kinase
LV: Left ventricular
LVPWd: The LV posterior wall end-diastolic thickness
LVPWs: The LV posterior wall end-systolic thickness
WGA: Wheat germ agglutinin
PVDF: Polyvinylidene difluoride membrane
CFs: Cardiac fibroblasts
